# Effects of Barefoot and Minimalist Footwear Strength-Oriented Training on Foot Structure and Function in Athletic Populations: A Systematic Review

**DOI:** 10.3390/jcm14217629

**Published:** 2025-10-28

**Authors:** Celia Rodríguez-Longobardo, Miguel Ángel Gómez-Ruano, Lorena Canosa-Carro

**Affiliations:** 1 Social Sciences of Physical Activity, Sport and Leisure Department, Faculty of Physical Activity and Sport Sciences, Universidad Politécnica de Madrid, 28040 Madrid, Spain; celia.rlongobardo@upm.es; 2 Department of Physiotherapy, Faculty of Medicine, Health and Sports, European University of Madrid, 28670 Madrid, Spain; lorena.canosa@universidadeuropea.es

**Keywords:** intrinsic foot muscles, extrinsic foot muscles, foot training, athletes

## Abstract

**Background/Objectives:** The popularity of barefoot and minimalist footwear training has increased in recent years, yet its impact on foot strength, morphology, and functional outcomes remains unclear, particularly in strength-training contexts beyond running-focused studies. Although some biomechanical and anecdotal evidence exists, no systematic review has specifically addressed the effects of foot-specific strength training interventions performed barefoot or with minimalist footwear. This review aimed to evaluate the effects of barefoot and minimalist footwear strength training interventions on foot muscle structure, force production, and neuromuscular function in healthy and athletic adults. **Methods:** A systematic review was conducted in accordance with the PRISMA and PICOS guidelines (PROSPERO number CRD420251134329). Comprehensive database searches were performed in July 2025. Eligible studies included barefoot or minimalist strength interventions in healthy and sportive adults, assessing outcomes related to foot muscle morphology, strength, functional performance, or neuromuscular adaptations. Methodological quality was evaluated using the PEDro and MINORS scales. **Results:** Seven studies involving 213 participants met the inclusion criteria. Most interventions led to significant improvements in intrinsic and extrinsic foot muscle volume, medial arch function, toe flexor strength, and neuromuscular control. Adaptations were particularly evident in interventions combining strength, balance, and agility exercises over multiple weeks. However, heterogeneity in protocols and outcome measures limited comparability. Some studies reported morphological gains without proportional improvements in strength or function. **Conclusions:** Barefoot and minimalist strength training can elicit beneficial morphological and functional adaptations in the foot. Nevertheless, inconsistencies in study design, small sample sizes, absence of follow-up assessment and lack of standardized protocols highlight the need for high-quality research to guide training recommendations beyond running-focused populations.

## 1. Introduction

In recent years, barefoot training and the influence of footwear characteristics, particularly toe box width and heel-to-toe drop, have received growing attention among the scientific community, professional athletes, recreational exercisers, healthcare providers, and fitness professionals. This interest has emerged in parallel with a broader trend toward natural or “minimalist” approaches to training, which emphasize reducing external support to promote intrinsic muscular and neuromotor adaptations [1,2].

Nevertheless, the majority of published research has centered primarily on running or walking biomechanics [3,4]. Numerous studies have assessed how barefoot running or running in minimalist footwear alters gait mechanics, joint loading, and injury risk compared with conventional shoes. While these findings have expanded our understanding of locomotor biomechanics, they do not directly address how barefoot or minimalist conditions may influence outcomes in non-running contexts [1,3,5].

An emerging area of interest is the potential for barefoot or minimalist training to enhance foot strength, morphology, and stability, especially through exercises that target the intrinsic (IFM) and extrinsic foot muscles (EFM). These adaptations may have implications not only for running performance or injury prevention but also in other athletic and training contexts where foot function plays a critical role.

Injuries like plantar fasciitis or Achilles tendinopathy affect thousands of patients every year worldwide, and their prevalence and prevention have been linked to weakness in the core foot muscle and EFM [6,7]. The training of these muscles has also been linked to an increase in volume in these muscles involved in sports like football or basketball [8]. If the use of barefoot shoes or minimalist footwear were to improve IFM or EFM strength indirectly, it could therefore be a potential mechanism to reduce these specific injuries by increasing stability and stiffness, but at this time, research focusing on this topic is scarce.

Despite this gap, many trainers and coaches advocate for the use of barefoot or zero-drop footwear during strength-based exercises, often under the assumption that these conditions promote greater stability, proprioceptive feedback, and overall training effectiveness [9,10]. Such recommendations, however, largely stem from anecdotal evidence or extrapolations from running-related studies rather than systematic evaluations of strength-training outcomes [1]. To date, no comprehensive review has synthesized the available research on barefoot conditions in strength-oriented contexts, leaving practitioners and athletes with limited evidence-based guidance [4].

A further challenge in this area is the lack of consistent terminology. Phrases such as “barefoot shoes”, “minimalist shoes”, or “no-drop shoes” are often used interchangeably in both research and practice, potentially leading to confusion [11]. The variability in footwear design features—such as heel stack height, longitudinal flexibility, overall weight, and toe box width—compounds this issue, as these characteristics may differentially influence biomechanics and performance. Recognizing this need for clarity, a 2018 framework established standardized criteria to classify footwear along a minimalist index, offering a more objective basis for comparison. However, many experimental studies still fail to report these parameters in detail, limiting reproducibility and comparability across investigations [1,3,11,12].

Emerging evidence suggests that footwear selection can influence lower-limb biomechanics across a variety of exercise modalities beyond running. Studies have demonstrated measurable effects of barefoot or minimalist conditions during activities such as deadlifting, walking, vertical jumping, and other plyometric movements. These findings support the notion that training without traditional footwear alters movement patterns and mechanical demands. Nonetheless, it remains unclear whether these biomechanical differences lead to meaningful effects on strength training performance. Specifically, their impact on force production, balance, or neuromuscular adaptations has yet to be clearly demonstrated [13,14,15,16].

Given the growing popularity of barefoot and minimalist training practices, the absence of a systematic synthesis of the evidence represents a critical gap in the literature. A better understanding of how barefoot strength training influences functional performance, foot muscle strength, or neuromuscular adaptations would not only inform athletes and coaches but also guide clinicians, physiotherapists, and researchers seeking to optimize training interventions.

Therefore, this systematic review aims to answer the following question: What are the effects of barefoot and minimalist footwear strength training interventions on foot muscle structure, strength, and functional performance in healthy and athletic adults?

## 2. Materials and Methods

A systematic review following the Preferred Reporting Items for Systematic Reviews and Meta-Analyses (PRISMA 2020) until July 2025 (Supplementary Material). In addition to the database search, backward citation tracking was performed but no further studies meeting the inclusion criteria were identified through this method. Guidelines and the Patient, problem or population, Intervention, Comparison, control or comparator, Outcome(s), Study type (PICOS) criterion was carried out. The review was registered in the PROSPERO database (number CRD420251134329).

### 2.1. Selection of Studies and Search Methodology

The literature search was conducted in PubMed, Scopus, and Web of Science (WOS) databases on 25 July 2025. The search strategy was designed using combinations of terms related to barefoot or minimalist training (e.g., “barefoot training”, “minimalist shoes”, “zero-drop shoes”, “natural running shoes”) and strength-related activities (e.g., “strength training”, “resistance training”, “weightlifting”, “muscle strength”, “hypertrophy”). The search was conducted across multiple databases without any restrictions on publication date or language.

An example of the search string used in WOS was: (“barefoot training” OR “barefoot exercise*” OR “barefoot activity” OR “unshod” OR “barefoot condition*” OR “barefoot walking” OR “barefoot running” OR “minimalist footwear” OR “minimalist shoes” OR “minimal shoes” OR “zero-drop shoes” OR “toe shoes” OR “vibram” OR “natural running shoes” OR “saguaro”) AND (“strength training” OR “resistance training” OR “weight training” OR “weightlifting*” OR “weight lifting” OR “powerlifting” OR “fitness” OR “gym-based training” OR “free weight*” OR “squat” OR “deadlift” OR “barbell training” OR “power training” OR “muscle strength” OR “strength” OR “hypertrophy”).

Studies were eligible for inclusion if they involved healthy, physically active, or trained adults (≥18 years) and included implemented resistance or strength training interventions performed barefoot or with minimalist footwear or applied specific strength training protocols targeting IFM and/or EFM. Studies that contained strength-oriented components regardless of the type of strength training were also included. Both experimental and observational studies were considered, provided they reported outcomes related to foot muscle strength, morphology, functional performance, or neuromuscular adaptations. Studies were excluded if participants had musculoskeletal pathologies in the lower limb, diabetes, obesity, were pregnant, if the intervention was performed exclusively with conventional footwear, involved sedentary individuals, children, orthotic or passive rehabilitation devices, or lacked relevant foot-related outcome measures. Non-original publications such as systematic or narrative reviews, meta-analyses, editorials, commentaries, conference abstracts, or other forms of gray literature were also excluded.

Study selection was conducted independently by two reviewers with academic backgrounds in Sports Rehabilitation and Exercise Science and specific expertise in systematic reviews. Both reviewers screened articles separately using pre-established inclusion and exclusion criteria, remaining blinded to each other’s decisions during the process. Disagreements were resolved through discussion until consensus was reached. The entire screening process was managed using Rayyan, a web-based platform designed for collaboration in systematic reviews [17].

### 2.2. Data Extraction

A standardized data extraction sheet was developed to systematically collect relevant information from each included study. The following variables were recorded: (i) author and year of publication; (ii) study design; (iii) study aim; (iv) participant characteristics; (v) presence or absence of a control group; (vi) description of the intervention protocol; (vii) outcome assessment methods; (viii) main results; and (ix) conclusions. One reviewer carried out the initial data extraction, and a second reviewer independently verified the completeness and accuracy of the entries. Any disagreements were resolved through consensus.

### 2.3. Risk of Bias Assessment

To assess the risk of bias in the included studies, different tools were applied based on study design. For experimental studies that included both an intervention and a control group, the PEDro scale (Physiotherapy Evidence Database) was used, which is a validated tool for evaluating methodological quality in clinical trials. Studies with a total PEDro score of 0–3 are considered ‘poor’, 4–5 ‘fair’, 6–8 ‘good’, and 9–10 ‘excellent’, considering that for complex interventions a total score of 8/10 is optimal [18]. For studies that included only a single intervention group without a control group, the Methodological Index for Non-Randomized Studies (MINORS) was applied to assess methodological quality. Every item receives a score between 0 and 2, where 0 means it was not mentioned in the article, 1 means it was mentioned but not sufficiently, and 2 means it was mentioned and sufficiently. For non-comparative research, the optimal overall score would be 16, while for comparative studies, it would be 24 [19]. Both tools were applied independently by two reviewers, with discrepancies resolved through discussion.

## 3. Results

### 3.1. Study Selection

**Figure 1:**
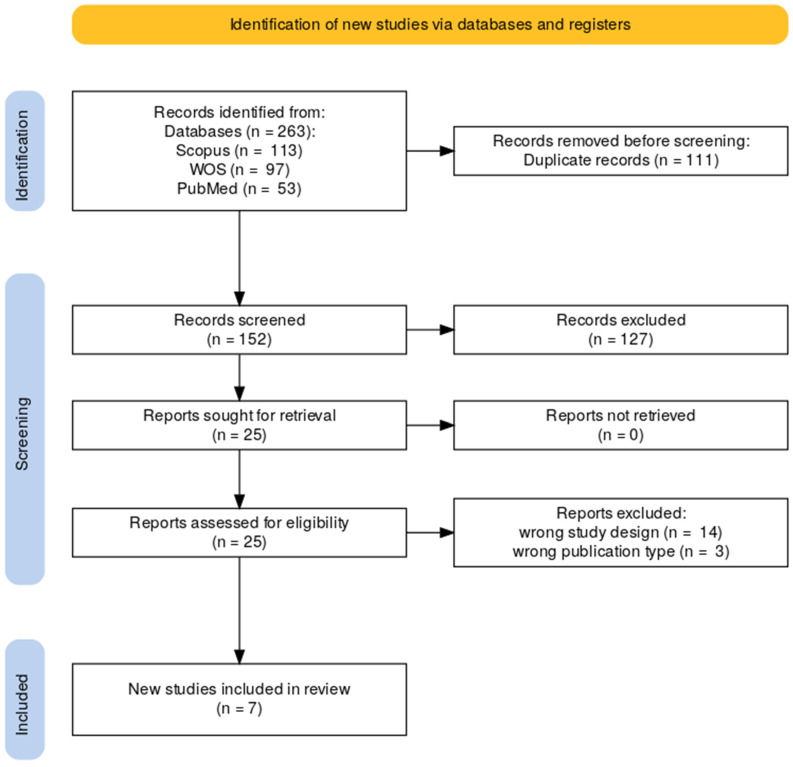
PRISMA flow diagram of the selected studies.

A total of 263 records were initially identified through database searching. After removing 111 duplicates, 152 titles and abstracts were screened. Of these, 25 full-text reports were sought for retrieval and successfully obtained. Following full-text assessment, 17 articles were excluded due to incorrect study design (*n* = 14) or publication type (*n* = 3). Ultimately, 7 studies met the eligibility criteria and were included in the qualitative synthesis. The study selection process is detailed in Figure 1.

### 3.2. Characteristics of Included Studies

The information regarding the characteristics of the 7 selected studies is presented in Table 1.

A total of 213 participants were included across the studies (72 males and 141 females), with a pooled mean age of approximately 27.8 ± 4.7 years. The age data from one study [20] was not available and thus excluded from this calculation. The majority of participants were recreational runners, with smaller groups of students and gymnasts. More specifically, the populations studied included recreational runners [5,20,21,22], sports science students [23], a mixed group of gymnasts and cheerleaders [24], and a group of habitually shod runners [25]. Intervention durations ranged from 3 weeks to 6 months, except for the gymnast and cheerleader group, who were habitually trained barefoot with no specific intervention applied.

**Table 1:** Data extraction of the selected articles.

Author, Year	Study Design	Aim	Participants	CG	Intervention
Chen et al., 2016 [19]	Experimental design	To examine how minimalist running shoes affect IFM and EFM volume in habitual shod runners, and how compliance influences these changes.	38 runners (21 males, 17 females) 34.8 ± 6.0 years old Running experience ~5.83 ± 5.11 years Running volume 34.44 ± 21.46 km/week EG: 20 CG: 18	Yes	6-month self-monitoring program comprising transition exercise regimens (calf strengthening exercise, balance training, and foot placement drills).
Khowailed et al., 2015 [5]	Single-group experimental design	To investigate how a 6-week simulated barefoot running program affects running kinetics in habitually shod female recreational runners.	12 female runners Training 3–5 days/week Running volume ~25 km/week 25.7 ± 3.4 years old	No	6-week training program of simulated barefoot running that included 15–20 min of running form drills, proprioceptive exercises, flexibility exercise, strengthening exercises (foot intrinsic, doming and hopping drill, toe grabs, and single-leg calf raises) and plyometric activities (single-leg hops, squat jumps).
Ridge et al., 2024 [22]	Cross-sectional	To compare foot strength, muscle size, and landing kinetics in gymnasts, cheerleaders, and controls during barefoot and shod landings.	48 females: 16 gymnasts, 16 cheerleaders, 16 non-athletes ~20.5 ± 1.4 years old	Yes	Habitual barefoot training (gymnasts); no intervention.
Shen et al., 2022 [18]	Experimental design	To investigate how a 12-week gait retraining program combined with foot core exercises affects arch structure, muscle strength, and movement dynamics.	26 male recreational runners Running volume ~29.1 ± 3.64 km/week EG: 13 CG: 13	Yes	12 weeks forefoot strike running and foot core exercises where participants progressed from double-leg heel raises on flat ground to single-leg raises on a step, with gradual increases in sets and repetitions. IFM exercises (towel curls, doming, and toe spreading) followed a similar volume progression (2 × 10 to 4 × 25 reps), with added load (0.25–0.5 kg) in later stages. Balance board exercises increased by 5 s weekly during the first 3 weeks and by an additional 5 s and/or one set during the final three weeks, while foot relaxation was maintained at 1 × 30 s throughout the protocol.
Goldmann et al., 2013 [21]	Experimental design	To assess how high-intensity athletic training performed in minimal footwear impacts toe flexor muscle strength.	47 healthy, not strength-trained female sport students 24 ± 5 years old EG: 18 Training control group (TG): 18 CG: 11	Yes	3 weeks, 5 days/week, of high intensity athletic training based on strength and agility tasks. Training program consisted of running, sprinting and jumping drills (e.g., zig-zag run, the direction changes, sprinter-ABC, one- and two-legged rope skipping, cutting maneuvers, one- and two-legged standing vertical and horizontal jumps, running upstairs and downstairs, and slalom racing). Chosen to increase weekly push-offs, totaling approximately 5000–6000 intensive push-offs over 15 sessions, performed at maximal effort.
Taddei et al., 2020 [20]	Experimental design	To examine how a foot training program influences muscle structure, strength, and running biomechanics in healthy recreational runners.	28 healthy recreational long-distance runners 41.8 ± 6.7 years old Running experience ~8.55 ± 7.41 years Running volume ~29.65 ± 11.46 km/week EG: 14 CG: 14	Yes	8 weeks of a IFM strengthening training program, with exercises such as feet tapping, forefoot ascend, invert/evert asymmetric, foot abduction, toes and ankle flexion, grabbing, holding and squeezing a ball, squeezing toe separators, toes abduction/adduction, short foot exercises, and plantar arch raise. Training volume generally ranged from one to three sets of 10–40 repetitions (or 20–40 s per set), with progressive overload achieved through gradual increases in the number of sets, repetitions, time under tension, postural demand (from seated to standing and single-leg positions), and, in some cases, resistance (e.g., elastic bands or increased object hardness). Progression was individualized based on participants’ ability to complete the exercises without pain, cramping, or loss of balance.
Koyama, 2022 [23]	Single-group experimental design	To examine how 12-week barefoot exercise training affected the foot’s arch height, muscle thickness, and strength.	14 young, healthy, habitually shod subjects (11 males, 3 females) 20.8 ± 1.1 years	No	12 weeks barefoot exercise training (3 days/week, 60 min sessions) consisting of: 1. Agility: ladder and mini-hurdle drills, including variations of jogging, step running, hopping, cross steps, zigzag sidesteps, and lateral movements; performed in forward and lateral directions using both ladders and mini-hurdles. Ladder drills: 4 sets with 1 min rest between types; mini-hurdle drills: 3 sets with 1-min rest between types. 2. Balance ball: using a BOSU (both-sides-up) device, standing with both feet on the flat platform while maintaining stability over the inflated dome (3 × 2 min, 1 min rest). 3. Strength exercises: standing calf-raises (3 × 60, 1 min rest) and the towel-gathering exercise, performing toe flexion at the metatarsophalangeal joint to pull a towel weighted to 60% of their maximum toe flexor strength, using only their toes (3 × 30, 20 s rest).
Author, year	Assessment		Results		Conclusions
Chen et al., 2016 [19]	MRI segmentations using Mimics software were analyzed on the right foot and leg at baseline and after 6 months to measure normalized IFM and EFM volumes.		Regarding volume, EG had significant increases in EFM (+7.05%, *p* = 0.01, d = 0.62) and IFM (+8.80%, *p* < 0.01, d = 0.54), mainly in the forefoot (*p* < 0.01, d = 0.64); CG showed no changes (*p* = 0.33–0.95, d = −0.08 to 0.21). MRS compliance averaged 39.2% (SD = 27.0); positively correlated with leg muscle volume change (r = 0.51, *p* = 0.02), with a non-significant trend for foot muscle volume (r = 0.39, *p* = 0.09).		Runners accustomed to traditional footwear showed increased IFM and EFM volumes after 6 months of transitioning to minimalist shoes. Increases in leg muscle volume were linked to how consistently participants used the minimalist shoes.
Khowailed et al., 2015 [5]	EMG of the tibialis anterior (TA) and the lateral gastrocnemius (GAS). Ground reaction forces (vertical impact peak, active peak, vertical instantaneous loading rate, and vertical average loading rate.		After 6 weeks of simulated barefoot running, participants showed reduced TA EMG activity (*p* < 0.001), increased GAS pre-activation, lower impact forces and loading rates, shorter stride length, step duration, and flight time, and increased stride frequency compared to shod running.		Motor pattern adaptations in habitually shod runners can occur within 6 weeks of simulated barefoot running. This led to reduced TA activity, potentially lowering injury risk. Neuromuscular changes required a habituation period, as early exposure did not produce the same effects.
Ridge et al., 2024 [22]	Foot muscle size, using ultrasound of: flexor digitorum brevis, quadratus plantae, abductor hallucis, fibularis brevis, fibularis longus, and tibialis posterior. Maximal toe flexion force generation. Ground reaction force through single foot drop landing trials shod (Nike Cheer Unite) and barefoot, in random order.		Gymnasts showed higher pVGRF (~15%) and faster time to stability (37%) and TTpVGRF compared to non-athletes; both gymnasts and cheerleaders stabilized faster than controls, with no difference between footwear conditions. Gymnasts and cheerleaders had greater total foot muscle size than controls (+24% and +14%, respectively), and only cheerleaders showed greater lateral toe-flexion strength (+40%) than controls.		Footwear reduces initial peak ground reaction forces but does not affect stabilization time across groups. Differences in landing kinetics and muscle size/strength were observed, with gymnasts and cheerleaders stabilizing faster—likely due to greater foot muscle size. Gymnasts showed the highest pVGRFs, which may raise injury risk, suggesting a need for muscle strengthening or reduced high-impact repetitions.
Shen et al., 2022 [18]	Arch morphology through manual palpation, toe flexion strength using a modified dynamometer, metatarsophalangeal joint (MPJ) flexors strength using a customized strength tester, arch kinematics and arch stiffness using Vicon motion analysis.		After 12 weeks, the EG showed a 5.1% increase in normalized navicular height (*p* = 0.027), significant gains in hallux flexion (20.5–21.7%, *p* = 0.001), and MPJ flexor strength (30.7–32.5%, *p* = 0.006). Arch kinematics also improved, with a 5.1% decrease in maximum arch angle and a 32.1% increase in arch height at touchdown (*p* < 0.001).		The combined 12-week gait retraining and foot core exercise program effectively improved arch structure and strength in both static and dynamic conditions, with moderate to large effect sizes. It is recommended for runners with weak arch muscles to enhance foot function.
Goldmann et al., 2013 [21]	Toe flexor strength was measured using a custom-made dynamometer that recorded joint moments during maximal voluntary isometric contractions (MVIC) at the MPJ, with force calculated via a strain-gauge load cell. Toe box bending moments were measured using a custom device that dorsiflexed the shoe at the MPJ to six angles (5–40°), while a load cell recorded resistance forces; moments were calculated by multiplying force by a fixed lever arm.		The flexible shoe had 6–8 times lower toe box bending moments than the traditional shoe. After training, MPJ moments significantly increased in the EG and training group (TG) at 0° MPJ dorsiflexion (*p* < 0.01 and *p* < 0.05), with no change in the CG. At 25° MPJ dorsiflexion, only the EG showed significant strength gains (*p* < 0.01), which were greater than both CG and TG (*p* < 0.05).		3 weeks of athletic training with minimal footwear significantly increased toe flexor strength (up to 20%), suggesting benefits for performance and injury prevention. Strengthening foot muscles through footwear choice may help reduce metatarsal stress and fatigue.
Taddei et al., 2020 [20]	Hallux and toe isometric strength, IFM anatomical cross-sectional area and volume (MRI), MLA range of motion (ROM) and stiffness, foot function scores, and propulsive impulses during running (vertical and antero-posterior).		After 8 weeks, the EG showed significant increases in IFM volumes (ABH +22.3%, ABV +12.1%, FDB +8.8%, FHB +17.7%; *p* < 0.05), but no significant improvement in toe flexor strength compared to controls. No changes were found in foot function (FHSQ), arch kinematics (MLA ROM or stiffness), or most running biomechanics. However, vertical impulse increased significantly in the EG (*p* = 0.021), and muscle volume was positively correlated with vertical impulse (*p* < 0.05).		The foot exercise protocol led to significant increases in IFM volume and vertical propulsive forces, but did not produce significant changes in toe flexor strength, arch kinematics, or foot function.
Koyama, 2022 [23]	Maximum voluntary isometric toe flexor strength using a toe grip dynamometer, maximum voluntary isometric plantar flexor strength with a digital force transducer, foot arch height, ultrasound of the IFM.		After 12 weeks of barefoot training, TFS, rTFS, PFS, and rPFS increased significantly by 32.7%, 29.1%, 48.8%, and 45.3%, respectively, with no change in FAH. Muscle thickness increased significantly in FDB (17.9%), QP (27.3%), FHB (10.3%), and total intrinsic muscles (13.2%), while ABH remained unchanged. No significant correlations were found between strength gains and changes in FAH or muscle thickness.		The toe flexor muscles’ capacity to generate force was enhanced by a 12-week barefoot training program.

ABH (Abductor hallucis), ABV (Abductor brevis), CG (Control group), d (Cohen’s d, effect size), EG (Experimental group), EFM (Extrinsic Foot Muscles), EMG (Electromyography), FAH (Foot Arch Height), FDB (Flexor digitorum brevis), FHB (Flexor hallucis brevis), FHSQ (Foot Health Status Questionnaire), GAS (Lateral gastrocnemius), IFM (Intrinsic Foot Muscles), MLA (Medial Longitudinal Arch), MPJ (Metatarsophalangeal Joint), MRI (Magnetic Resonance Imaging), MVIC (Maximal Voluntary Isometric Contraction), pVGRF (Peak Vertical Ground Reaction Force), PFS (Plantar Flexor Strength), QP (Quadratus plantae), rPFS (Relative Plantar Flexor Strength), rTFS (Relative Toe Flexor Strength), SD (Standard Deviation), TFS (Toe Flexor Strength), TG (Training Group), TTpVGRF (Time to peak Vertical Ground Reaction Force).

Regarding outcome assessments, foot muscle morphology was evaluated via MRI or ultrasound. Both IFM and EFM were assessed in two studies [21,24], while only IFM was measured in the other two articles [22,25], and only EFM in one study [5]. Foot strength was evaluated in five studies [20,22,23,24,25], while functional assessments of the foot (e.g., stability, impact absorption, or arch dynamics) were included in four studies [5,20,22,24].

The interventions analyzed across the selected studies aimed to improve foot muscle strength, morphology, and function through barefoot or minimalist conditions. While the specific protocols varied—from isolated IFM strengthening to combined balance, agility, and plyometric exercises—all emphasized strengthening tasks performed without conventional footwear. Several studies incorporated progressive, multi-week training programs [5,20,22,23,25] while others examined the impact of long-term habitual barefoot activity [24]. Despite methodological differences, the interventions consistently aimed to challenge the foot musculature under load or instability.

Overall, the studies consistently reported positive adaptations in foot muscle morphology and function following barefoot or minimalist strength training interventions. Significant increases in IFM and EFM volumes, particularly in the forefoot region, were observed [21], alongside improvements in toe and metatarsophalangeal joint flexor strength in interventions involving strength, agility, and foot-core exercises [20,23,25]. A simulated barefoot running program, with strength exercises, led to neuromuscular and biomechanical adaptations, including reduced tibialis anterior activity, greater gastrocnemius pre-activation, and more efficient stride patterns [5]. Gymnasts and cheerleaders, habituated to high-impact exercises and landing drills, showed larger foot muscle cross-sectional area and faster stabilization than non-athletes, regardless of footwear condition [24]. Additional improvements were noted in medial arch mechanics and navicular height [20], while increases in vertical impulse were associated with greater IFM volume following targeted training [22]. However, it is important to notice that not all interventions led to significant gains in toe flexor strength or functional foot performance despite morphological changes [22], and some morphological adaptations were not significantly correlated with strength outcomes [25]. These inconsistent findings were present in those studies with a more briefly intervention or with interventions performing lower intensity exercises.

### 3.3. Risk of Bias Results

All studies evaluated using the PEDro scale demonstrated methodological quality ranging from good to excellent (Table 2). The most common limitation across these trials was the absence of blinding of participants and therapists, which is an expected challenge in exercise-based interventions. Despite this, the overall design, randomization, and reporting procedures in these studies support the reliability of their findings.

Regarding the non-randomized studies, methodological quality was assessed using the MINORS scale (Table 3). Khowailed et al. and Ridge et al. [5,22] included comparison groups and were therefore evaluated with the comparative version of the tool (maximum score = 24), obtaining scores of 14/24 and 16/24, respectively, indicating moderate quality. In contrast, Koyama et al. [23] lacked a comparison group and were evaluated using the non-comparative version (maximum score = 16), receiving a score of 10/16. Notably, none of the three studies reported elements such as prospective sample size calculation, follow-up duration, or information on loss to follow-up, which limits the robustness of their conclusions.

## 4. Discussion

This systematic review aimed to examine the effects of barefoot and minimalist strength training interventions on foot muscle structure, strength, and functionality in trained and healthy adults. Overall, the evidence seems to support that targeted barefoot or minimally shod strength interventions can lead to structural and functional adaptations in the foot.

A consistent finding across studies was the increase in muscle volume, particularly within IFMs such as the flexor digitorum brevis, quadratus plantae, and flexor hallucis brevis, as well as EFMs like the gastrocnemius and soleus, when assessed [21,22,25]. Notably, forefoot regions exhibited the most prominent hypertrophic adaptations [21]. These findings align with previous meta-analytic evidence indicating that IFM training improves muscle morphology, balance, and navicular height, particularly through exercises like the short foot exercise [26].

Improvements in muscle strength were also frequently observed. Several studies reported increased toe flexor and metatarsophalangeal joint strength following interventions that included not only isolated foot-core exercises but also strength and agility drills performed barefoot [5,23,25]. These functional gains were generally aligned with improvements in medial arch kinematics, such as greater navicular height and reduced arch angle at touchdown [20]. However, it is important to note that not all interventions resulted in improved force production. One study reported muscle hypertrophy without a concomitant increase in toe flexor strength or functionality [22], suggesting a potential dissociation between structural and functional adaptations. This might guide coaches, trainers and physical therapist when prescribing exercises for improving foot function; this review seems to indicate that interventions under 8 weeks or those interventions focusing only in specific strength training of the IFM and EFM rather than including more broad exercises, with higher intensity and functional exercises might be less effective in improving foot strength.

In terms of biomechanical and neuromuscular outcomes, the study involving simulated barefoot running paired with foot-strengthening exercises [5] demonstrated favorable adaptations in stride parameters and lower limb muscle activation. These included reduced tibialis anterior activity, increased gastrocnemius pre-activation, and more efficient gait mechanics, highlighting the systemic impact of combined foot-focused training. This suggests that incorporating foot-specific strength work into running-related interventions may enhance not only local adaptations but also global movement patterns.

The observational study involving gymnasts and cheerleaders [24] highlighted notable long-term adaptations from regular athletic training. Gymnasts, who train barefoot, showed superior neuromuscular control and foot function, including higher peak vertical ground reaction force and faster stabilization time than non-athletes. Both gymnasts and cheerleaders stabilized faster than controls regardless of footwear condition, suggesting functional improvements from sport-specific training. Morphologically, both groups had greater foot muscle size, with cheerleaders also showing +40% greater lateral toe-flexor strength. These findings suggest that consistent exposure to athletic tasks involving foot engagement enhances foot musculature and function, but training barefoot may provide additional neuromuscular and morphological advantages, particularly in parameters related to impact control and IFM development.

One thing to point out is that most existing research in this area has focused on running populations, with an emphasis on gait efficiency, impact forces, and injury risk during barefoot running [1,27]. While informative, this focus has left strength-based barefoot interventions in other sports and general fitness contexts relatively underexplored. This review is the first, to our knowledge, to systematically compile evidence on barefoot strength interventions across multiple types of training modalities and populations.

Moreover, there is a lack of standardized protocols regarding volume, load, and progression of barefoot strength training. Studies differed widely in duration, intensity, and the type of exercises prescribed. Recommendations from Malheiros de Souza et al. (2023) [26] suggest progressing IFM training from seated to standing positions and using specific volume guidelines to optimize adaptation. Yet, most included studies did not report in detail how progressive overload or individualized load adjustments were implemented. In order to build a stronger body of knowledge regarding this topic, future studies need to address the footwear characteristics using the minimalist index [11] as well as defining the load, times, repetitions and sets for the exercise programs. It would also be positive to move into more measurable type of exercises from the ones that are commonly used to like the “towel grabbing” or the short foot exercise where measuring the force and the load that is being used is fairly complicated.

Furthermore, the majority of studies focused on IFMs, with only a few addressing the role of EFMs in arch support and foot stabilization [21]. Given that EFMs like the gastrocnemius, tibialis posterior, and fibularis contribute significantly to foot and ankle function [27] future interventions should consider their targeted inclusion. Additionally, many commonly used IFM exercises (e.g., towel curls, toe spread, short foot) are widely studied, but few trials address how compound movements, performed barefoot, affect foot strength or injury prevention. Future research should explore the effects of movements like squats and deadlifts, which are known to heavily engage EFMs like the gastrocnemius and tibialis posterior while barefoot, affect these muscles volume, strength and function.

In line with this, another important gap in the literature is the lack of longitudinal or interventional studies evaluating barefoot execution of gym-based strength training exercises (e.g., barbell squats or deadlifts). While some observational studies have compared acute biomechanical differences between performing these exercises barefoot versus wearing shoes [16,28], these investigations are limited to single-session assessments and do not explore the long-term effects on foot musculature, strength, or injury risk. Consequently, there is a lack of evidence regarding how regular barefoot resistance training influences foot function in adults who habitually perform these compound lifts without footwear.

An important consideration when interpreting the current evidence is the methodological rigor of the included studies. Although trials evaluated with the PEDro scale demonstrated acceptable overall quality, recurring weaknesses were identified across designs. The absence of participant and therapist blinding in randomized studies reflects a broader challenge in exercise-based interventions, where the nature of physical exercise interventions typically makes it unfeasible to blind participants or therapists, as they are inherently aware of the training being delivered or received. Meanwhile, non-randomized studies assessed by the MINORs scale consistently lacked critical elements such as prospective sample size justification, monitoring of drop-out rates, or adequate follow-up periods, which are factors that limit reproducibility and raise concerns about internal validity. Due to the qualitative and descriptive nature of the synthesis, no formal assessment of publication bias (e.g., funnel plots or Egger’s test) was performed. These gaps highlight the need for more robust trial designs in future research, particularly when assessing interventions that demand longitudinal tracking of morphological and functional adaptations.

### Limitations

Some limitations should be considered when interpreting the findings of this review. The number of included studies was small, with relatively modest sample sizes, which limits the generalizability of the findings. The heterogeneity of interventions in terms of duration, intensity, and exercise type restricted direct comparisons across studies. Methodological shortcomings were also noted, including limited blinding and incomplete reporting of footwear characteristics. Finally, most studies focused on recreational runners rather than strength-trained populations, leaving important gaps regarding barefoot training in gym-based or sport-specific contexts.

## 5. Conclusions

This systematic review synthesized the current evidence on the effects of barefoot and minimalist training interventions on foot muscle morphology, strength, and functional outcomes in healthy and athletic adults. The findings suggest that training in barefoot or minimalist conditions can promote structural adaptations, particularly increases in IFM and EFM volume, and may enhance functional outcomes such as medial arch mechanics, stability, and neuromuscular efficiency. Improvements were most consistently observed in interventions involving progressive, multi-week programs that incorporated a variety of strength, agility, higher intensity and balance exercises. This evidence is mainly focused on IFM. Overall, this review supports the growing perspective that barefoot and minimalist training can serve as an effective strategy to strengthen foot musculature and improve certain functional outcomes.

Despite these promising results, the evidence also indicates that morphological adaptations may not consistently translate into proportional gains in muscle strength or functional performance, especially in short-duration or low-intensity interventions. Furthermore, while barefoot or minimalist training appears to positively influence neuromuscular control and impact absorption, the long-term implications for injury prevention, athletic performance, and strength training in gym-based contexts remain unclear.

Moreover, the heterogeneity of study designs, small sample sizes, and limited focus on strength-based exercise contexts highlight the need for further high-quality research to establish clear, evidence-based recommendations for athletes, clinicians, and coaches focusing on the specifics of minimalist shoes, the exercises and its load as well as creating longer interventions that can fully show the effects of barefoot training.

## 6. Future Directions

Despite promising evidence on the benefits of barefoot and minimalist training for foot muscle morphology and function, current research remains limited in scope and generalizability. Most studies have focused predominantly on recreational runners, leaving a substantial gap in our understanding of how barefoot strength interventions might affect athletes from other disciplines. Future research should investigate the effects of barefoot or minimalist training in sports where foot strength, proprioception, and ground contact mechanics play a critical role, such as martial arts, gymnastics, dance, or Olympic weightlifting.

A particularly relevant gap lies in the absence of experimental studies assessing barefoot or minimalist strength training in gym-based environments. Research is needed to examine the effects of barefoot strength training on foot muscle hypertrophy, structural integrity, and injury prevention in individuals who regularly perform high-load or compound movements without traditional footwear.

To build a more robust evidence base, future studies should adopt longitudinal designs with well-controlled variables such as intensity progression, exercise specificity, and frequency. Including functional outcome measures—such as arch stiffness, balance, or kinetic chain integration—will help determine the practical impact of barefoot strength training on both performance and injury prevention.

Lastly, future research should expand the scope of analysis beyond the IFM to include EFM like the tibialis posterior, gastrocnemius, and fibularis. Incorporating tools such as EMG, imaging, and kinetic/kinematic assessments would allow a more comprehensive understanding of the neuromuscular adaptations that occur in response to barefoot training modalities.

## Figures and Tables

**Table 2:** Risk of bias results using the PEDro scale.

	Study			
PEDro Item *	Chen et al., 2016 [19]	Shen et al., 2022 [18]	Goldmann et al., 2012 [21]	Taddei et al., 2020 [20]
1	yes	yes	yes	yes
2	Yes	yes	yes	yes
3	Yes	yes	no	yes
4	yes	yes	yes	yes
5	yes	no	no	no
6	no	no	no	yes
7	yes	yes	no	yes
8	yes	yes	yes	yes
9	yes	yes	yes	yes
10	yes	yes	yes	yes
11	yes	yes	yes	yes
Total score	9	9	7	10

* PEDro item: 1, eligibility criteria; 2, random allocation to groups; 3, concealed allocation; 4, similar groups at baseline; 5, blinding of subjects; 6, blinding of therapists; 7, blinding of assessors; 8, measures of key outcomes obtained from >85% of the subjects; 9, treatment received or analyzed by intention to treat; 10, between-group comparison results reported; 11, point measures and measures of variability provided. PEDro scores of 0–3 are considered “poor”, 4–5 “fair, 6–8 “good” and 9–10 “excellent”.

**Table 3:** Risk of bias results using the MINORS scale.

	Study		
MINORS Scale Item *	Khowailed et al., 2015 [5]	Ridge et al., 2024 [22]	Koyama, 2022 [23]
1	2	2	2
2	2	2	2
3	2	2	2
4	2	2	2
5	0	2	2
6	0	0	0
7	0	0	0
8	0	0	0
9	0	0	0
10	2	2	0
11	2	2	0
12	2	2	0
Total score	14 ^1^	16 ^1^	10 ^2^

* MINORS Scale Item: 1, clearly stated aim; 2, inclusion of consecutive patients; 3, prospective collection of data; 4, endpoints appropriate to the aim; 5, unbiased assessment of the study’s endpoint; 6, follow-up period appropriate; 7, loss to follow up less than 5%; 8, study size calculation prospectively; 9, control group; 10, contemporary groups; 11, baseline equivalence of groups; 12, adequate statistical analysis. ^1^ scores evaluated with the comparative version of the tool (maximum points = 24); ^2^ scores evaluated with the non-comparative version of the tool (maximum points = 16). Regarding the comparative version of the tool, a total score of 16–22 indicates a low risk of bias, 8–15 indicates a moderate risk of bias, and 0–7 indicates a high risk of bias.

## Data Availability

No new data were created.

## Supplementary Materials

The following supporting information can be downloaded at: https://www.mdpi.com/article/10.3390/jcm14217629/s1, PRISMA 2020 Checklist [29].

## Abbreviations

The following abbreviations are used in this manuscript:

EFM	Extrinsic Foot Muscles
IFM	Intrinsic Foot Muscles
WOS	Web of Science
